# Rheumatic Valve Disease Presenting As Mitral Stenosis and Regurgitation

**DOI:** 10.7759/cureus.86671

**Published:** 2025-06-24

**Authors:** Nismat Javed, Rosanna Pineda, Vikram Itare, Shoaib Ashraf, Gayathri Kamalakkannan

**Affiliations:** 1 Internal Medicine, BronxCare Health System, Icahn School of Medicine at Mount Sinai, New York City, USA; 2 Internal Medicine, BronxCare Health System, New York City, USA; 3 Cardiology, BronxCare Health System, Mount Sinai Morningside, New York City, USA; 4 Cardiovascular Disease, BronxCare Health System, New York City, USA

**Keywords:** mitral regurgitation, mitral stenosis, prognosis, rhd, treatment

## Abstract

Rheumatic heart disease (RHD) affects many people globally, with the majority of cases in low- and middle-income countries. The disease often leads to significant valve damage, requiring surgical intervention due to complications such as regurgitation or stenosis. Although new cases have declined in high-income countries due to improved control of rheumatic fever (RF), chronic RHD remains a concern. Limited data on RHD in the US highlights the need for improved understanding and management. This case report discusses a 49-year-old male with worsening dyspnea, productive cough, and bilateral leg swelling who was diagnosed with severe mitral stenosis and regurgitation secondary to RHD. Diagnostic imaging revealed significant valve dysfunction and thrombi in the left atrial appendage, as confirmed by both echocardiography and a transesophageal echocardiogram. Despite initial management with medications and anticoagulation, the patient was transferred to a tertiary center for potential mitral valve intervention. This case highlights the importance of early identification and multimodal imaging in managing RHD, as well as the ongoing debate regarding the efficacy of mitral valve repair versus replacement.

## Introduction

Rheumatic heart disease (RHD) has affected over 33 million people globally, mainly in low- and middle-income countries [1]. The disease causes valve damage, often requiring surgery due to regurgitation or stenosis. In developed countries, chronic RHD persists from childhood infections, but new cases are rare due to better RF control [1]. Mostly, RF is caused by an autoimmune response to untreated group A streptococcal infection and progresses to RHD [2]. Rheumatic heart disease most often targets the mitral valve structurally and can lead to regurgitation as a complication, where acute rheumatic valvulitis initially causes mitral regurgitation [3,4]. Rheumatic mitral stenosis, globally the leading cause of mitral stenosis, gradually narrows the orifice, enlarges the left atrium, drives pulmonary hypertension, and promotes atrial fibrillation. In contrast, mitral regurgitation, the most common rheumatic lesion, predominates in younger patients. With age, fibrosis and calcification can accumulate, leading to stenosis. Contemporary ACC/AHA guidance emphasizes the use of three-dimensional echocardiography for detailing valve morphology, quantifying severity, and determining candidacy for intervention [3,4]. Recent studies on pathology further show that this valvular dysfunction reverberates through both left- and right-heart structures, heightening risks of heart failure, pulmonary hypertension, and systemic embolism, making early recognition, comprehensive imaging, and timely treatment essential [5]. Additionally, data in the US regarding RHD is limited. While RHD has decreased rates of mortality, the mortality rates from other valvular diseases have increased with time [6]. Timely identification of the disease can improve prognosis and optimize management. With this rationale in mind, we discuss the case of a 49-year-old patient who was eventually diagnosed with rheumatic valve disease.

## Case presentation

A 49-year-old African-American male with a past medical history of asthma presented to the ED with the chief complaints of gradually worsening dyspnea on exertion, productive cough, and bilateral lower leg swelling for the past two weeks. He noticed that his exercise tolerance had been reduced to 25-30 steps daily instead of the usual five blocks. The patient also had a history of smoking (three to four cigarettes per day).

**Figure 1:**
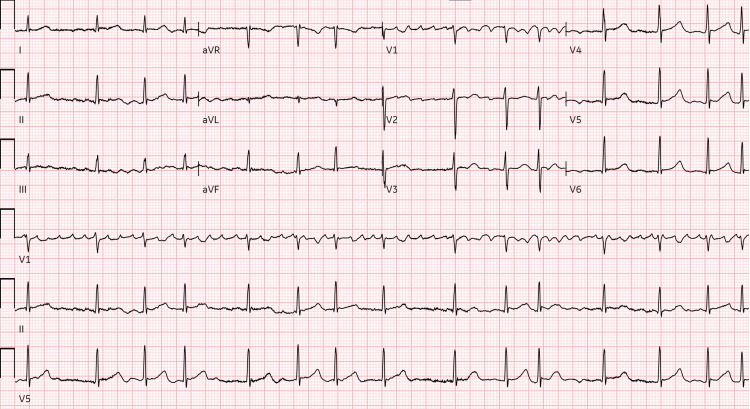
Electrocardiogram showing atrial fibrillation with rapid ventricular response

In the ED, the patient was hypertensive (162/111 mm Hg). Initial EKG showed atrial fibrillation with RVR (Figure 1).

The physical exam revealed crackles in both lung fields and bilateral leg edema. A systolic murmur was also heard over the left sternal border. He was started on metoprolol 5 mg, furosemide 40 mg, and Lovenox 100 mg in the ED and admitted for further management. The lab investigations are presented in Table 1.

**Table 1:** Lab investigations

Investigation	Result	Normal range
Hemoglobin (g/dl)	14.0	12.0-16.0
White blood cells (/uL)	7100	4800-10,800
Platelets (/uL)	291,000	150,000-400,000
Sodium (mEq/L)	137	135-145
Potassium (mEq/L)	4.3	3.5-5.0
Calcium (mEq/L)	8.8	8.5-10.5
Chloride (mEq/L)	100	98-108
Glucose (mg/dl)	103	70-120
Bicarbonate (mEq/L)	23	24-30
Blood urea nitrogen (mg/dl)	17	6-20
Creatinine (mg/dl)	1.1	0.5-1.5

**Figure 2:**
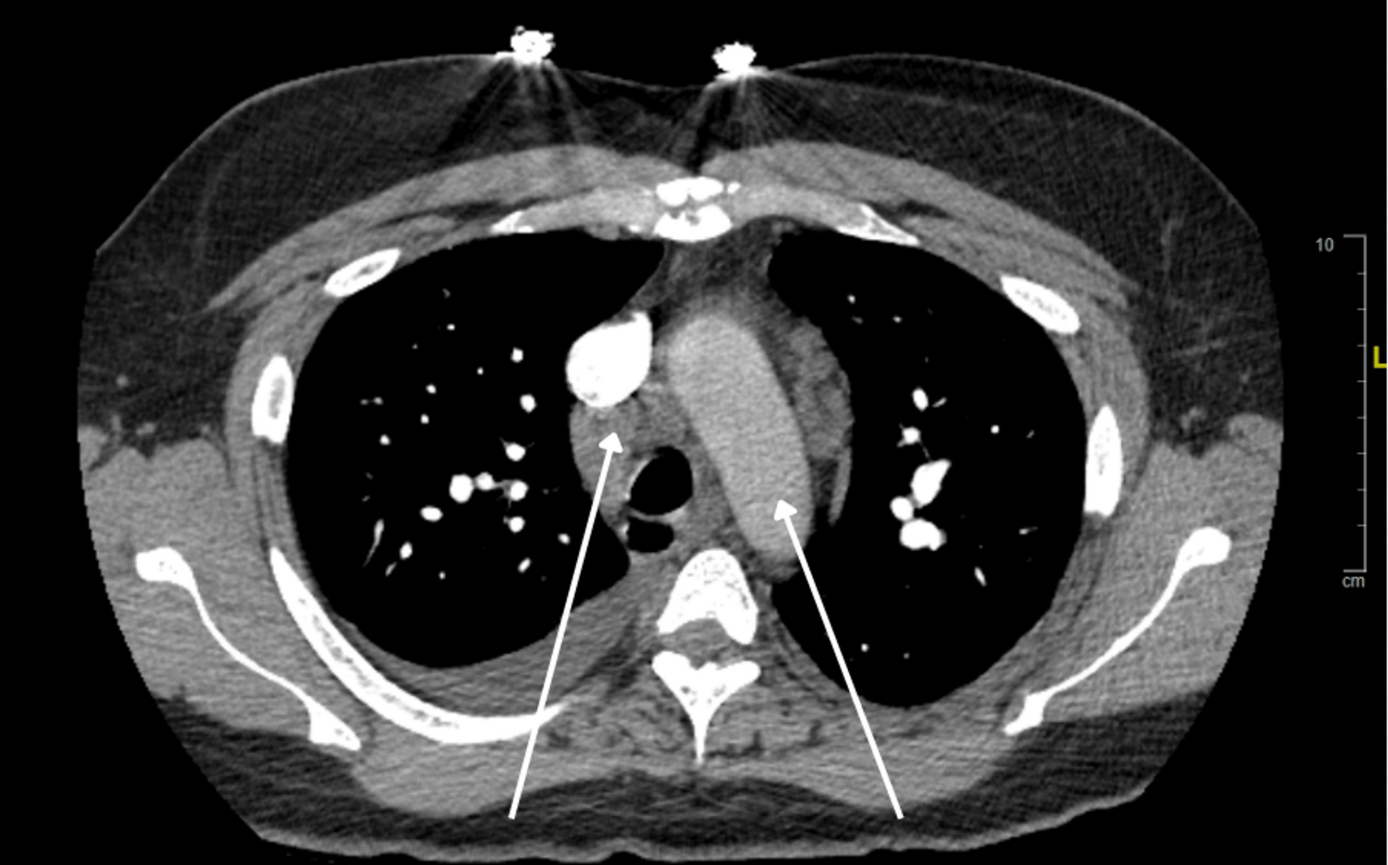
CT chest with contrast without evidence of pulmonary embolism Findings labelled by white arrow

The patient's thyroid function tests were normal, and the urine drug screen was negative. A CT scan of the chest with contrast was performed, which ruled out a pulmonary embolism (PE) (Figure 2). 

**Figure 3:**
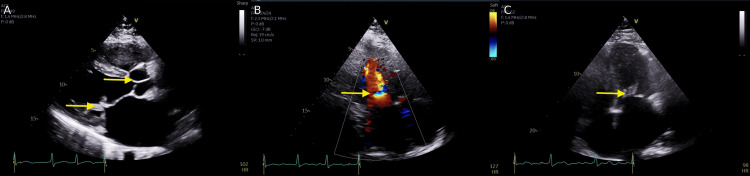
Parasternal long axis view of echocardiogram showing stenotic orifice of mitral valve (A). Apical 4-chamber view showing regurgitant jet on colour Doppler (B) and stenotic mitral valve on similar view (C) Findings labelled by yellow arrow

The echocardiogram revealed a reduced ejection fraction (EF) of 43.51%. There was evidence of concentric left ventricular hypertrophy (LVH) with global wall motion abnormalities. The patient also had abnormal septal motion consistent with right ventricular volume and pressure overload. It also showed a moderately dilated left atrium, dilated right atrium and ventricle, moderate mitral stenosis, and mitral regurgitation with rheumatic valve changes (Figure 3). The findings were also consistent with right ventricular dysfunction, and the patient had trace pulmonic regurgitation. 

Right and left cardiac catheterization were performed the next day, which ruled out any ischemic disease. The angiogram revealed patent coronary arteries with luminal irregularities (Table 2). The pulmonary artery pressures and wedge pressure were elevated, consistent with volume overload. 

**Table 2:** Right heart catheterization values (in mmHg)

Site	Pressures
Pulmonary capillary wedge (PCW)	19
Pulmonary artery (PA)	57/32 (mean 41)
Right ventricle (RV)	39/7
Right atrium (RA)	2/2 (mean 1)

**Figure 4:**
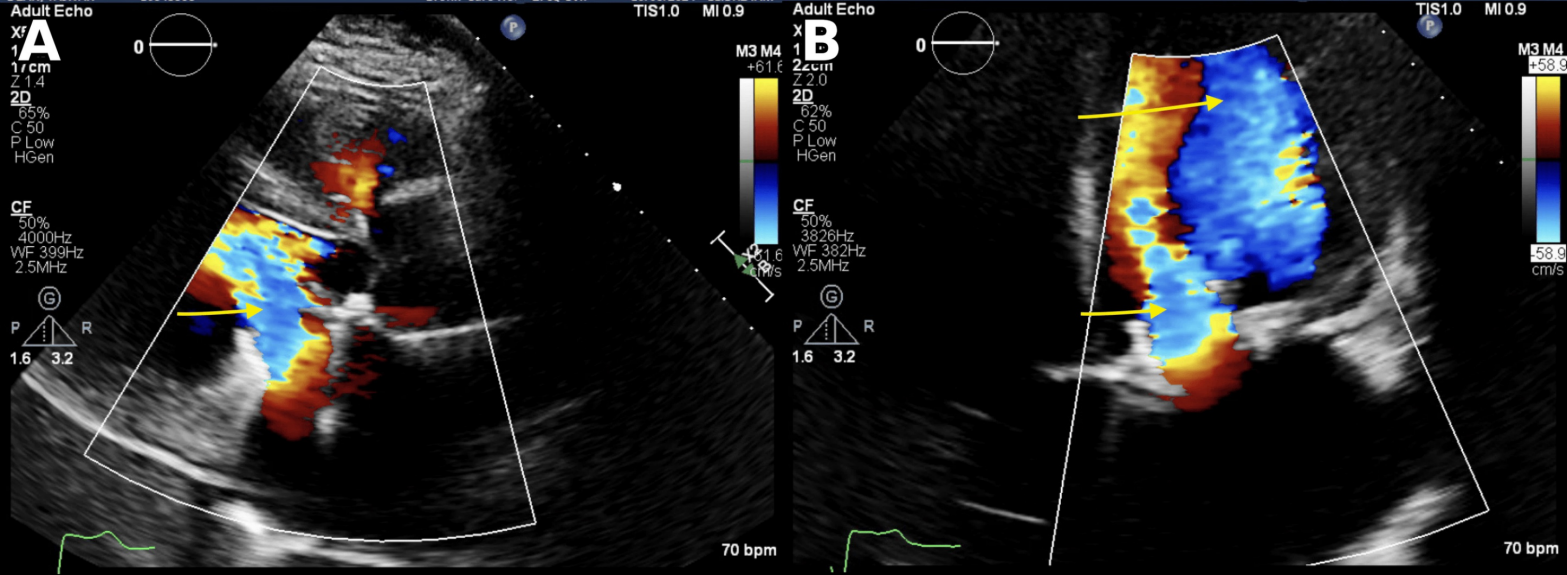
Transesophageal echocardiogram showing rergurgitant jet at mitral surface in parasternal long axis view (A) and posteriorly directed eccentric jet shown in apical view (B) Findings labelled by yellow arrows

He underwent a transesophageal echocardiogram, revealing a rheumatic mitral valve and severe mitral regurgitation with a posterior jet. The patient had an effective regurgitant orifice area of 0.55 cm² and a regurgitant volume of 72 mL, consistent with severe mitral regurgitation. It also revealed a severely dilated left atrium and decreased velocities in the left atrial appendage. Two small thrombi were also noted in the left atrial appendage (Figure 4).

Cardioversion was not performed because of the presence of thrombi. After an interdisciplinary team discussion, he was deemed a candidate for mitral valve intervention and was transferred to a tertiary center. Infectious diseases was also consulted regarding antibiotic prophylaxis, and the patient was administered intramuscular benzathine penicillin. He did well post-operatively after mitral valve replacement with a biatrial maze procedure and left atrial appendage occlusion with a clip. He was followed up in outpatient settings.

## Discussion

Mitral stenosis can develop from multiple causes. It is often a result of acute rheumatic fever, even in patients without a known history of the fever, likely due to missed diagnosis. In developed countries, mitral stenosis is typically diagnosed around age 50 when symptoms emerge, whereas in developing countries, it manifests much earlier. The progression of the disease varies by region, reflecting differences in healthcare quality and local epidemiology [7].

The pathophysiology of RHD remains poorly understood. The process involves calcium deposition, similar to atherosclerosis, which is initiated by oxidized lipids and inflammation, resulting in the formation of fibrocalcific plaques over time [7]. Some cases progress rapidly, necessitating early intervention during the acute phase, while others develop slowly, resulting in chronic complications. Valve damage, particularly involving mitral regurgitation and stenosis, varies between patients, often resulting in inflammation, scarring, and prolapse of valve leaflets. Aortic regurgitation commonly occurs due to scarring of the cusps. The resulting valve dysfunction may require repair or replacement to break the cycle of progressive damage and regurgitation [8].

Calcification of the mitral annulus in the elderly can cause significant obstruction by limiting diastolic annular dilation and restricting leaflet movement, leading to mitral stenosis in the population [7]. Factors such as aging, hypertension, diabetes, atherosclerosis, systemic inflammation, and kidney disease contribute to calcific degeneration. Studies link mitral stenosis to stroke, atrial fibrillation, arrhythmias, atrioventricular block, and increased mortality [7]. Shared cardiovascular risk factors and inflammation play a role, with low fetuin-A levels, fibroblast growth factor, and high inflammation markers being implicated. Data on disease progression is limited, but studies show worsening mitral gradients and a declining survival rate in severe cases [7]. 

Echocardiography is the primary tool for assessing RHD, with the World Health Organization recommending it for screening in areas with high prevalence [9]. Echocardiography identifies both acute and chronic features of rheumatic valvulitis, such as the presence of stenosis or regurgitation, annular dilation, leaflet prolapse, and calcification in the mitral and aortic valves [9]. The mitral valve leaflet separation index is a useful marker for mitral stenosis, with international guidelines defining clinically significant mitral stenosis as a valve area of ≤1.5 cm² [9]. Current diagnostic criteria classify RHD into three categories: definite, borderline, or normal, based on morphological and Doppler findings [9]. Morphological features of rheumatic heart disease (RHD) affecting the mitral valve include thickening of the anterior leaflet (≥3mm during diastole, adjusted for age), thickening of the chordae, limited leaflet mobility, and excessive leaflet tip movement during systole [9].

Cardiac magnetic resonance imaging (CMR) is the reference standard for quantifying mitral regurgitation (MR) severity and its impact on cardiac remodeling when echocardiography is inconclusive, providing highly reproducible measurements of regurgitant volume and fraction, as well as left and right ventricular volumes and function [10]. The American College of Cardiology and the American Heart Association recommend CMR for MR quantification when transthoracic echocardiography is inadequate; however, they note that CMR is less helpful for a detailed assessment of mitral valve anatomy [11]. For mitral stenosis, CMR can provide planimetric assessment of the mitral valve area and evaluate chamber remodeling; however, echocardiography remains the primary modality [10]. Cardiac computed tomography (CT) is not routinely used for functional quantification of mitral regurgitation (MR) or mitral stenosis (MS), but it is valuable for anatomical assessment, including planimetric measurement of the mitral valve orifice and pre-procedural planning, especially in cases of heavy calcification or suboptimal echocardiographic windows [12-14]. 

Catheter-based measurements remain the most reliable method for assessing intracardiac and vascular pressures, valve gradients, chamber pressures, hemodynamic blood flow, stroke volume, and cardiac output, as well as directly measuring oxygen content and saturation in all chambers [15]. Cardiac catheterization is recommended when clinical findings from non-invasive methods differ, uncertainty exists about pulmonary artery pressure, valve disease severity is unclear (especially in multivalvular cases), or echocardiographic imaging quality is insufficient [15].

Surgical options include mitral valve repair or mitral valve replacement. Current guidelines recommend mitral valve (MV) repair over replacement for degenerative MV disease [16]. Although MV repair is also preferred for RHD, there is no consensus among experts on this approach. However, previous research in Taiwan has indicated the benefits of MV repair for treating infective endocarditis (IE); for example, 2.1% of patients with repair had infective endocarditis, compared to 2.8% of patients who underwent replacement [16]. Several large retrospective studies suggest better long-term outcomes with MV repair than replacement, and a systematic review has demonstrated improved short- and long-term event-free survival, as well as acceptable reoperation rates, for MV repair in RHD patients [17]. In a population-based study comparing real-world outcomes, no significant difference in all-cause mortality was found between MV repair and replacement during a six-year follow-up (33.4% vs. 32.5%) [17]. This suggests MV repair does not offer a survival advantage over replacement. Wilkins' score plays a role in determining candidates with a poor prognosis (score >8), who require early surgical repair, as observed in our patient with minimally mobile leaflets, extensive thickening, and calcification [18]. Additionally, patients undergoing MV repair at high-volume medical centers showed no better outcomes than those treated at regional or lower-volume hospitals [19].

In patients who only undergo mitral valve repair, persistent progression of RHD may lead to recurrence of valve dysfunction, necessitating redo valve interventions [17, 20]. The disease has a favorable prognosis when identified and treated early. When left untreated, disease progression tends to result in irreversible adverse outcomes.

## Conclusions

Mitral stenosis, often stemming from untreated or under-treated acute rheumatic fever, was diagnosed based on echocardiographic findings, indicating severe mitral regurgitation and stenosis, a dilated left atrium, and evidence of small thrombi. Despite intervention efforts and management, including anticoagulation and the prospect of surgical intervention, the chronic nature of RHD presents ongoing challenges as the valve damage continues to progress. While mitral valve repair is often preferred, there is no clear survival advantage over replacement, and reoperation rates remain a concern. The case highlights the importance of timely identification and the role of early intervention in effectively managing RHD.
